# Contrasting Proteomic and Metabolomic Responses of Bermudagrass to Drought and Salt Stresses

**DOI:** 10.3389/fpls.2016.01694

**Published:** 2016-11-11

**Authors:** Tiantian Ye, Haitao Shi, Yanping Wang, Fan Yang, Zhulong Chan

**Affiliations:** ^1^Key Laboratory of Plant Germplasm Enhancement and Specialty Agriculture, Sino-Africa Joint Research Center, Wuhan Botanical Garden, Chinese Academy of SciencesWuhan, China; ^2^University of Chinese Academy of SciencesBeijing, China

**Keywords:** abiotic stress, bermudagrass, carbon metabolism, metabolomic, photosynthesis, proteomic

## Abstract

Bermudagrass (*Cynodon dactylon*) is one of tolerant grass species to drought and salt. The comparative analyses of bermudagrass in response to drought and salt stresses at the physiological, proteomic, and metabolomic levels were performed in this study. The physiological results indicated that osmolytes accumulation, ROS level and antioxidant enzyme activities were extensively changed by drought and salt stresses. Through comparative proteomic analyses, we successfully identified a total of 77 proteins involved in photosynthesis, oxidative pentose phosphate, glycolysis, and redox metabolic pathways when exposed to drought and salt stresses. Among them, 36 proteins were commonly regulated by both treatments, while other 40 and 13 proteins were specifically regulated by drought and salt, respectively. Totally 15 proteins were involved in carbon metabolic pathway. Moreover, contents of 37 metabolites including amino acids, organic acids, sugars, and sugar alcohols were regulated by drought and salt treatments. Among them, 18 commonly modulated metabolites were involved in carbon and amino acid metabolic pathways. Drought treatment for 21 days caused less accumulation of sugars and sugar alcohols and increased ROS level in bermudagrass which led to relatively more severe cell membrane reflected by high EL-value and lower survival rate when compared to 400 mM salt treatment for 21 days. These results suggested that drought and 400 mM NaCl stresses for 21 days treatment affected common and specific changes in bermudagrass, which would provide new insights to understand the underlying molecular mechanisms and metabolic homeostasis of bermudagrass in responses to abiotic stresses.

## Introduction

Drought and salt are two major abiotic factors limiting plant growth and development, which induce various changes at different levels, such as morphological, physiological, proteomic, and metabolic levels (Zhu, 2002; Gibbs and Greenway, 2003). As one of the most important warm-season turfgrasses, bermudagrass (*Cynodon dactylon*) is widely used in the construction of golf courses, sport fields, and lawns, and wetland vegetation restoration (Shi et al., 2012, 2013a,b). Previous studies indicated that the bermudagrass exhibited tolerance to several abiotic stresses including drought and salt (Shi et al., 2012, 2013a,b, 2014b), and developed complex strategies such as physiological, proteomic, metabolic changes to cope with stress conditions. Shi et al. (2012) suggested that changes of water status, osmolyte accumulation and antioxidant defense system partly reflected the drought stress tolerance of bermudagrass varieties. Exogenous application of NO, H_2_S and polyamines enhanced the tolerances of bermudagrass to abiotic stresses such as drought, salt, osmotic and cold (Shi et al., 2013a,b, 2014b). In addition, salt, chilling, and submergence tolerances of some bermudagrass varieties might be correlated with variations of root and shoot responses (Qian et al., 1997), relative leaf water content (RWC) (Tan et al., 2010), dehydrin abundance (Hu et al., 2010), chlorophyll content, proline content, soluble sugars content, ethylene accumulation, reactive oxygen species (ROS) level and antioxidant enzyme activities (Lu et al., 2007; Bailey-Serres and Voesenek, 2010).

Recently, proteins changed by stress treatments were identified through proteomics approach in turfgrass. Zhao et al. (2011) identified 54 proteins associated with water-deficit tolerance in bermudagrass leaves. A total of 39 proteins showing different abundance in leaves and stems of two drought contrasting bermudagrass varieties (Yukon and Tifgreen) were found to be responded to drought stress, and these proteins were mainly involved in photosynthesis (PS), glycolysis, N-metabolism, tricarboxylicacid (TCA), and redox pathways (Shi et al., 2014a). In addition, 51 proteins enriched in redox, tricarboxylicacid cycle, glycolysis, photosynthesis, oxidative pentose phosphate pathway, and amino acid metabolisms were regulated by CaCl_2_ treatment in cold stressed bermudagrass leaves (Shi et al., 2014c). Metabolites including amino acids, organic acids, sugars, and sugar alcohols in bermudagrass leaves were commonly modulated by CaCl_2_ and cold treatments (Shi et al., 2014c).

Drought condition causes water deficit and osmotic stress in plants, while high salinity causes both an ionic and an osmotic stress (Zhu, 2002). Therefore, plants not only develop common signaling transduction pathways in response to drought and salt stress conditions, but possess some unique pathways to cope with drought and salt signaling. Several bermudagrass varieties showed relatively high tolerance to both drought and salt stress when compared to crop plants (Shi et al., 2013a,b). We hypothesized that the bermudagrass developed common and contrasting mechanisms to cope with drought and salt stresses. Although specific mechanisms of drought and salt tolerances have been investigated extensively in bermudagrass, limited information is available on the simultaneously comparative analyses of drought and salt stress responses at the proteomic and metabolomic levels. Therefore, investigation of the proteomic and metabolomic changes in responses to abiotic stresses in bermudagrass will be important to reveal different mechanisms involved in drought and salt stress responses and to improve plant stress tolerance.

In the present study, comparative analyses on proteomic and metabolomic responses of bermudagrass to different abiotic stresses were performed. The results showed that drought and salt stress treatments resulted in similar and contrasting changes in bermudagrass. These data would provide some novel insights to understand the molecular mechanisms and metabolic homeostasis of bermudagrass in response to drought and salt stresses. Identification of stress regulated proteins in bermudagrass would provide gene candidates to improve tolerance of bermudagrass and crop plants to abiotic stresses.

## Materials and methods

### Plant materials and experimental design

The bermudagrass Yukon seeds were kindly provided by American Seed Research of Oregon Company. After 3 days of stratification at 4°C in the dark, the seeds were sown in the flowerpot filled with soil in the greenhouse in Wuhan Botanical Garden, Chinese Academy of Sciences. The growth condition were maintained at 25 ± 2°C average temperature, 65–75% relative humidity, and 16 h light irradiance of about 150 μmol quanta m^−2^s^−1^ per day. The plants were irrigated with nutrient solution (18–18–18 of nitrogen–phosphorus–potassium per 100 g fertilizer, plus 3% magnesium and microelement) twice every week. Three-week-old bermudagrass healthy seedlings with nearly same crown size were used for the next drought and salt treatments.

A completely randomized design with two factors (drought and salt) was employed. For drought treatment, two different watering regimes were performed as follows: a well-watered treatment and a water deficit treatment (watering for 21 days and then drought for 21 days). For salt stress treatment, the concentration of NaCl was gradually increased to the final concentration 400 mM (adding 100 mM each day). The survival rate of stressed bermudagrass was recorded at 7 days re-watering after 21 days stress treatment. The leaf samples were collected at 0, 7, 14, 21 days after treatments for physiological indexes analyses. The leaf samples at 14 day subjected to control and stress conditions were harvested for proteomic and metabolomic assays based on measured electrolyte leakage data. The whole experimental design was displayed as Figure S1. For each independent experiment, every plant sample was extracted from at least 35 bermudagrass plants. All the experiments in this study were carried out three times at different time interval with three technical replications for each treatment.

### Determination of electrolyte leakage (EL) and relative leaf water content (RWC)

EL was determined according to the methods of Shi et al. (2012). In brief, the initial conductivity was determined after gently shaking at room temperature for 6 h at 150 rpm using a conductivity meter (Leici-DDS-307A, Shanghai, China). The fully releasing conductivity was measured after boiling at 121°C for 20 min using previous samples. The percentage of electrolyte leakage was determined as the ratio of the initial conductivity to fully releasing conductivity.

The RWC were determined according to the methods of Shi et al. (2012). The fresh weight (FW) was weighed immediately after collection, and the dry weight (DW) was quantified after incubation for 16 h at 80°C, and the RWC (%) was measured as (FW − DW)/FW × 100%.

### Measurement of proline content

Briefly, 0.25 g leaf samples were grinded to power and then extracted in 3% (w/v) sulfosalicylic acid for 10 min at 100°C. After that, 2 ml ninhydrin reagent and 2 ml glacial acetic acid were added to the 2 ml extraction solution. The mixed solution was boiled at 100°C for 40 min. After cooling to room temperature, the proline level of sample was determined by measurement of absorbance at 520 nm as described previously (Shi et al., 2012).

### Determination of malondialdehyde (MDA) content

The MDA content was extracted using thiobarbituric acid (TBA) reagent and boiled at 100°C for 20 min as previously described by Yang et al. (2010). After cooling to room temperature and centrifugation at 15000 g for 10 min, the MDA concentrations were determined at 450, 532, and 600 nm of absorbance with a spectrometer. The MDA concentration can be estimated through the following formula (μmol l^−1^) = 6.45 (A_532_ − A_600_) − 0.56A_450_ (Yang et al., 2010; Shi et al., 2012).

### Quantification of sucrose and soluble total sugars

The sucrose and soluble total sugars were determined according to the methods of Shi et al. (2012). The sucrose content and soluble total sugar content of samples were measured at 480 nm of absorbance and calculated by using the standard curve with known concentration of sucrose and glucose.

### Determination of ROS accumulation and antioxidant enzyme activities

The protein concentration was quantified according to the method of Bradford with bovine serum albumin (BSA) as the standard (Bradford, 1976). The concentration of H_2_O_2_ was determined as described by Shi et al. (2012). The H_2_O_2_ level was calculated according to a standard curve of H_2_O_2_. The ${\text{O}}_{2}^{•-}$ content was assayed by the plant ${\text{O}}_{2}^{•-}$ ELISA Kit based on antibody-antigen-enzyme-antibody complex following the manufacturer's instruction (Dingguo, Beijing, China). The absorbance was quantified at 405 nm.

The antioxidant enzyme activities of catalase (CAT), glutathione reductase (GR), and peroxidase (POD) were determined using CAT Assay Kit (S0051, Beyotime, China), GR Assay Kit (Beyotime, Shanghai, China), and plant POD Assay Kit (Nanjing Jiancheng Bioengineering Institute, Nanjing, China), respectively, according to the manufacturer's instructions.

### Protein extraction and 2-DE

Total protein was extracted according to the previously described method with slight modifications (Chan et al., 2007). Briefly, 1 g frozen powder from plant leave were homogenized extensively with 5 ml of pre-cooled homogenization buffer [20 mM Tris-HCl (pH 7.5), 1.05 M sucrose, 10 mM EGTA, 1 mM DTT, 1 mM PMSF and 1% (v/v) Triton X-100] on ice, and centrifuged at 10,000 g for 30 min at 4°C. The supernatant was then mixed with equal volume of Tris-HCl (pH 7.8) buffered phenol. After centrifugation at 10,000 g for 30 min at 4°C, the above phenol phase was mixed with five volumes of ice-cold saturated ammonium acetate in methanol overnight at −20°C. The total proteins were collected through centrifugation was stored at −80°C or dissolved in the lysis buffer [7 M urea, 2 M mithiourea, 4% (w/v) of 3-[(3-cholamidopropyl)-dimethylammo-nio]-1-propane sulfonate (CHAPS), 65 mM DTT and 0.2% (w/v) of carrier ampholyte (pH3.5–10)]. After dissolving extensively and centrifugation, the protein supernatant was quantified through the Bradford's method (Bradford, 1976).

The 2-DE was performed as described by Shi et al. (2013a) with minor modification. Briefly, 1 mg of total proteins was applied onto an immobilized pH gradient (IPG) strip (17 cm, pH 4–7, Bio-Rad, USA) and rehydrated extensively at room temperature overnight. The next day, the rehydrated strips were transferred to isoelectric focus (IEF) in the Protein IEF system (Bio-Rad, USA). The conditions of IEF and SDS-PAGE were the same as described by Shi et al. (2012).

### Gel image analysis and protein spot identification by MALDI-TOF-MS

The 2-D gels stained by Coomassie brilliant blue (CBB) R250 [containing 50% (v/v) of methanol, 15% (v/v) of acetic acid and 0.1% (w/v) of CBB-R250] were scanned using an EPSON PERFECTION V700 PHOTO scanner (Epson), and protein spots of 2-D gel with different abundance changes were analyzed using PDQuest 2-DE Analysis Software (BIO-RAD, USA). The protein spots (fold change ≥2) were used for trypsin digestion and MALDI-TOF-MS analysis with AXIMA-CFR plus (Shimadzu Biotech, Kyoto, Japan) as reported by Li et al. (2012) and Shi et al. (2013a). MASCOT software (Mascot Wizard 1.2.0, Matrix Science Ltd., http://www.matrixscience.com) was used to analyze the MS data. Since bermudagrass is an un-sequenced species, the homologous proteins were blasted against sequenced plant species. In the searching process against NCBInr and Swiss-Port protein sequence databases, peptide masses were assumed to be monoisotopic, and 100 ppm was used as mass accuracy, and one missing cleavage site was the maximum, and modifications were also considered. The minimum score of 43 and the minimum sequence coverage of 6% in MOWSE analysis were used to keep the confidence of the identification results.

### Quantification of metabolites

The metabolites extraction and derivatization were performed as described by Lisec et al. (2006) and Sanchez-Villarreal et al. (2013). Briefly, 100 mg of plant leaves was ground in liquid nitrogen, and 1.4 mL of 100% methanol (pre-cooled at −20°C) was then added and vortexed for 10 s. After that, 60 μL of 0.2 mg /mL Ribitol was added as an internal quantitative standard and vortexed for 10 s. After shaking for 10 min at 70°C in a thermomixer, the mixed solution was centrifuged at 11,000 g for 10 min. The supernatant was transferred to a new tube, and 750 μL chloroform and 1.4 mL ddH_2_O were added to the sample. After vortex, the sample was centrifuged at 2200 g for 15 min. Then 150 μL supernatant was transferred into a 1.5 mL tube, and 40 mL methoxyamination reagent was added. The sample was mixed well and shaken at 37°C for 2 h before 70 μL MSTFA reagent and time standards (alkane mixtures) were added. The sample was shaken at 37°C for another 30 min. Derivatization reaction of an empty reaction tube was also prepared as a control. Then the metabolites were analyzed using GC-TOF-MS (Agilent 7890A/5975C, CA, USA) according to the procedure of Lisec et al. (2006) and Shi et al. (2014c).

For GC-TOF-MS, 1 μL of derivatizated sample was injected into a DB-5MS capillary (30 m × 0.25 mm × 0.25 mm, Agilent J&W GC Column, USA), and was analyzed by an Agilent 7890 gas chromatograph with a model 5975 mass spectrometer (Agilent Technologies, Palo Alto, CA, USA) according to the procedure of Lisec et al. (2006) and Shi et al. (2014c). The inlet temperature was set at 260°C. After a solvent delay for 6 min, initial GC oven temperature was set at 60°C; after injection for 1 min, the GC oven temperature was raised to 280°C with 15°C/min, and held at 280°C for 15 min finally. The injection temperature was set to 280°C and ion source temperature was adjusted to 230°C. Helium was used as the carrier gas with a constant flow rate set at 1 mL/min. The measurement was performed with electron impact ionization (70 eV) in the full scan mode (m/z from 30 to 600). Metabolite Data Processing and Analysis Peak finding, peak integration, and retention time correction were performed with the Agilent MSD Productivity Chemstation software. The metabolites were identified based on retention time index specific masses, via comparing with reference spectra in mass spectral libraries (NIST 2005, Wiley 7.0). After metabolite identification, quantification of metabolites was performed based on the pre-added Ribitol in the process of metabolite extraction that was used as an internal standard. For the derivatization, an empty reaction tube was prepared as a blank control, and also a C10, C12, C15, C19, C22, C28, C32, and C36 n-alkane mixtures were used for the determination of RIs to improve the accuracy of qualitation. The data acquisition was done in multiple batches with one batch including one replicate for all treatments that was to minimize run order effects. Three biological replicates and two technical replicates, totally six replicates were used for the metabolomics analysis.

### Cluster and metabolic pathway analyses

Hierarchical cluster analysis was performed using CLUSTER program (http://bonsai.hgc.jp/~mdehoon/software/cluster/) (de Hoon et al., 2004), and resulting tree figures were displayed using the software package and Java Treeview (http://jtreeview.sourceforge.net/) as Shi et al. (2013a) described. The proteins showing significant abundance changes were classified using the Classification SuperViewer Tool (http://bar.utoronto.ca/ntools/cgi-bin/ntools_classification_superviewer.cgi) (Provart and Zhu, 2003), and functional categories of every protein were assigned using MapMan (http://mapman.mpimp-golm.mpg.de/general/ora/ora.html) (Thimm et al., 2004). The pathway graph of carbon metabolism was obtained from KEGG (http://www.genome.jp/kegg/pathway.html). Normalized frequency (NF) of each functional category was assayed as sample frequency of each category in this experiment/background frequency of each category in genome.

### Statistical analysis

All the experiments in this study were repeated three times, and the data shown are the means ± SEs. For each independent experiment, plant sample was extracted from at least 35 bermudagrass plants. SPSS 13.0 software was used for statistical analysis. Analyses of variance (ANOVA) for variables from measurements were used for testing the species and treatment differences according to Duncan's method. Different letters above the columns in each figure indicate significant differences at *P* < 0.05.

## Results

### Comparison of morphological and physiological responses to drought and salt

**Figure 1:**
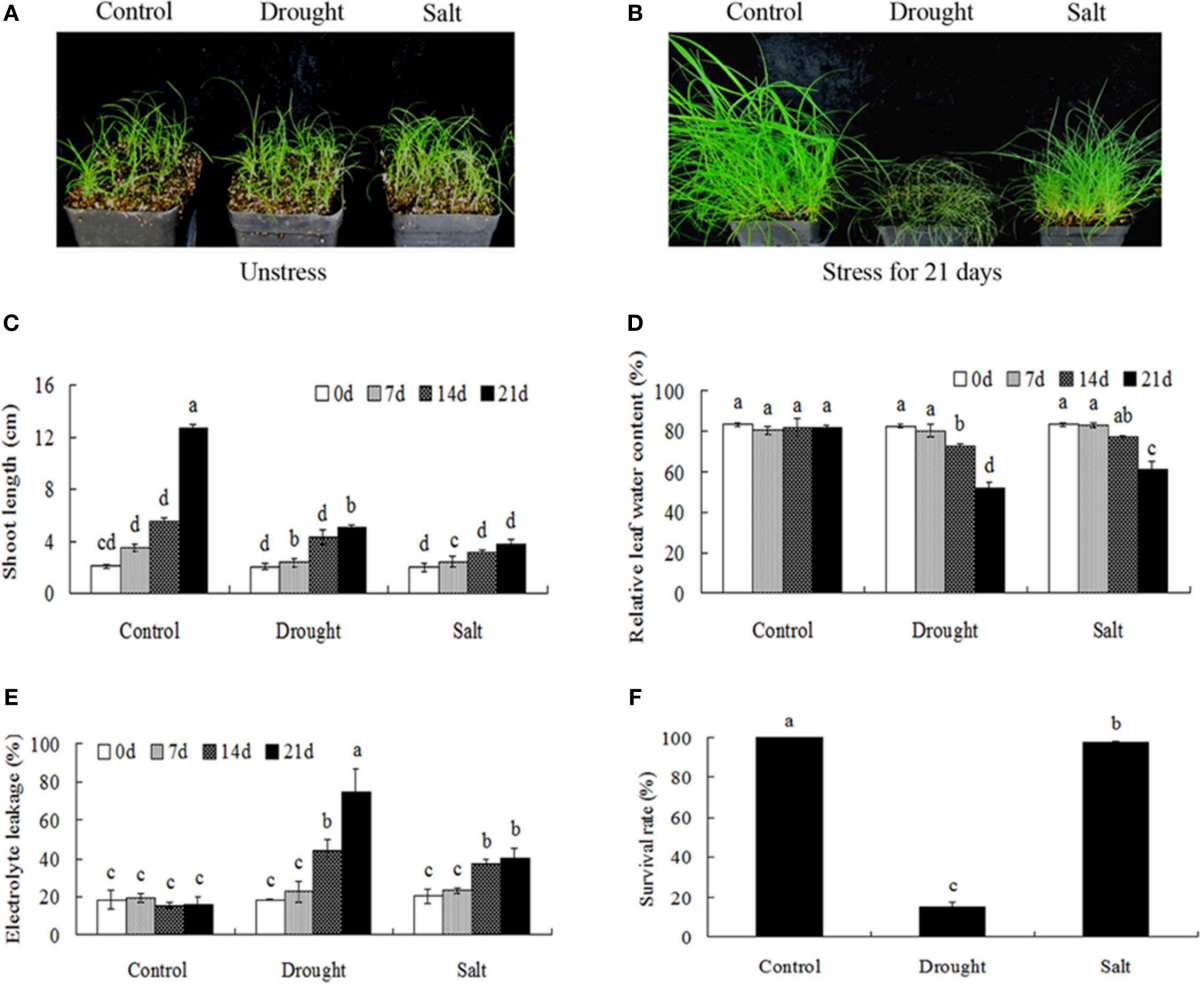
**Comparison of physiological responses to drought and salt in bermudagrass. (A)** The photograph showing 3-week-old Bermudagrass that subjected to control condition and stress conditions for 21 days. Shoot length **(B)**, RWC **(C)**, chlorophyll content **(D)**, EL **(E)** of Bermudagrass under unstress and stress condition at designated time intervals. **(F)** Survival rate of Bermudagrass after 21 days of control and stress treatments. The data represent the means of three independent experiment ± SE, and data followed by different letters are significantly different from each other at *P* < 0.05 according to Duncan's method.

Drought and salt stresses had imposed significant effects on the growth and development of bermudagrass. When exposed to 21-days drought and salt treatments, significant differences in the growth of bermudagrass were observed. Drought stress caused serious damages (growth inhibition, drooped leaves) on bermudagrass with short shoot, whereas salt stress had little effect on plant growth (green leaves) (Figures 1A–C). Drought and salt stresses progressively decreased the RWC with the duration of the treatment (Figure 1D). However, drought and salt stresses progressively increased the EL with the duration of the treatment. Additionally, drought treatment imposed more serious damage to leaves in terms of EL during the 14–21 days in comparison to salt stress (Figure 1E). Accordingly, drought stress had serious effects on the survival rates of bermudagrass, while the salt stress had less effect on plant survival (Figure 1F).

### Regulation of osmolytes in bermudagrass under drought and salt conditions

**Figure 2:**
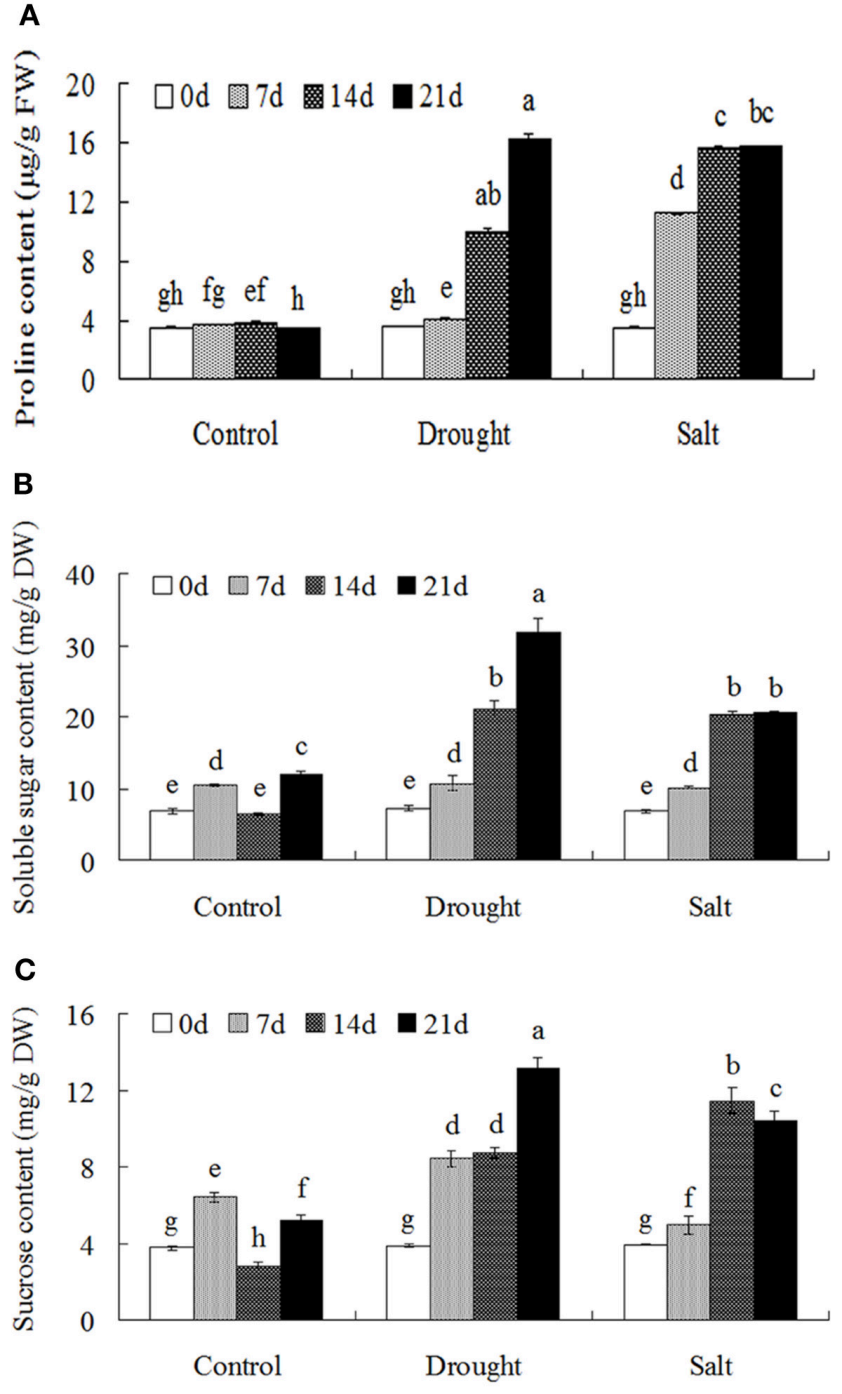
**Osmolytes accumulation of bermudagrass that subjected to unstress and stress conditions at designated time intervals**. Changes of proline content **(A)**, soluble sugars **(B)** and sucrose content **(C)** of bermudagrass during under control and stress conditions at indicated days. The results shown are means ± SE (*n* = 4), and the results followed by different letters are significantly different from each other at *P* < 0.05 according to Duncan's method.

Drought and salt stress treatments largely induced proline accumulation with the duration of the treatments (Figure 2A). In addition, the contents of soluble sugars and sucrose were largely increased after drought and salt treatments (Figures 2B–C).

### Comparative proteomic analyses of bermudagrass in response to drought and salt stresses

**Figure 3:**
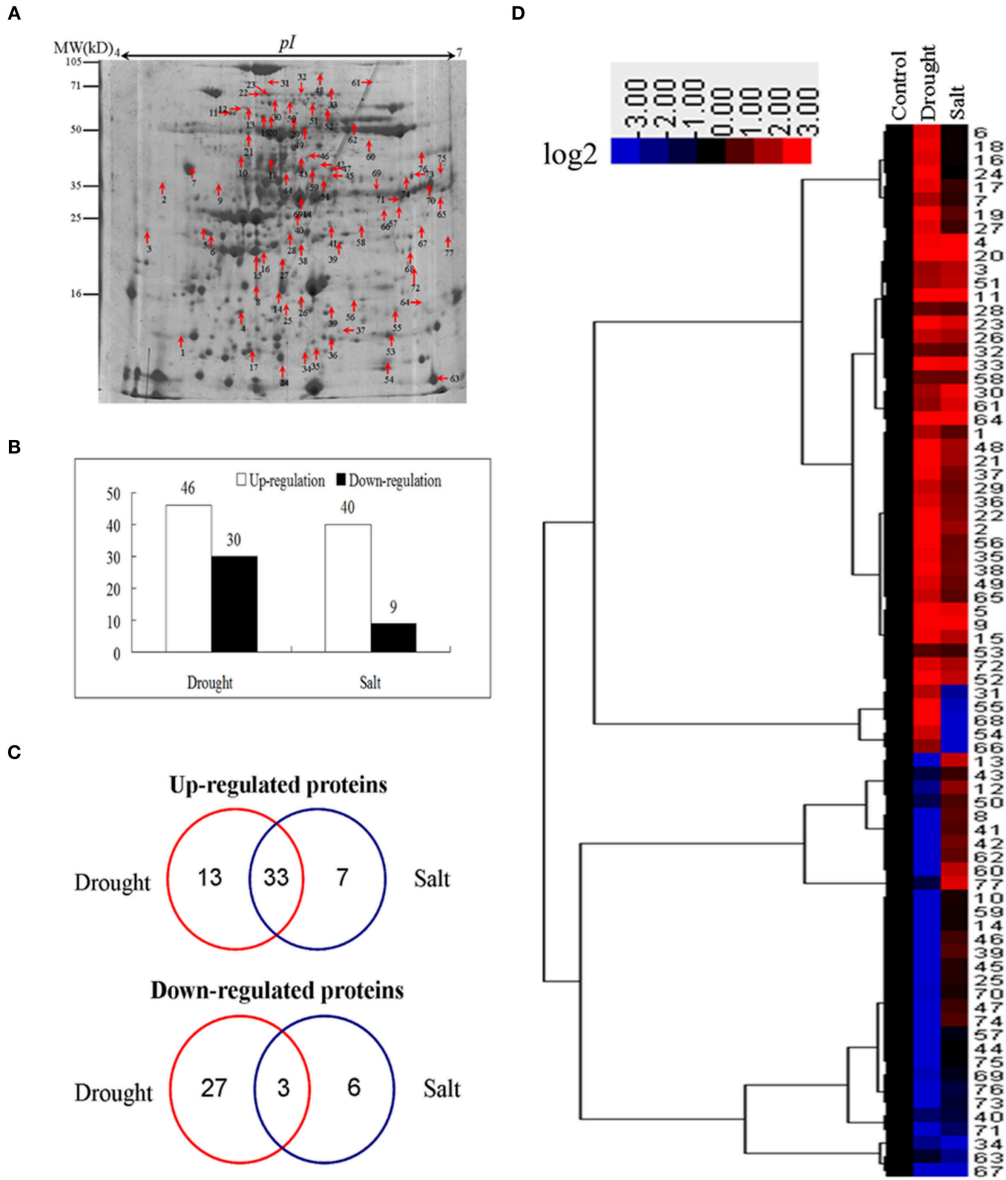
**Cluster analysis and comparative distribution of co-regulated proteins by drought and salt treatments. (A)** A sketch map to show proteome patterns of bermudagrass in responses to drought and salt. The protein spots induced at least two folds by drought and salt were marked with arrows as shown. Proteins were separated in the first dimension on the IPG strip (pH 4−7), and in the second dimension on 12.5% SDS-PAGE. **(B)** The number of proteins up and down regulated by drought and salt treatments. **(C)** Hierarchical cluster analysis of fold change of coregulated proteins by drought and salt treatments. Resulting tree figure was displayed using the software package and Java Treeview. **(D)** Venn diagram showing the number and of proteins that overlapped among three types of drought and salt.

To identify proteins of bermudagrass simultaneously affected by drought and salt treatments, proteomic analyses based on 2-DE were performed using 14 days stressed samples which showed about 50% EL (Figure 1D). Generally, more than 300 spots were reproducibly detected on all gels. Compared to controls, in total 105 proteins were significantly (2-fold change) affected by at least two types of stress treatment (Figure 3A), and 77 of 105 proteins were successfully identified via MALDI-TOF-MS (Table 1). Among 77 proteins, abundances of 76 and 49 proteins were changed by drought and salt treatments, respectively. The MS results were matched against NCBInr and Swiss-Port protein sequence databases using MASCOT software, and the best matched protein with high confidence score was selected as the final result of each protein spot (Table 1 and Table S1). Although Viridiplantae (Green Plants) was chosen as taxonomy during Mascot database search, most putatively identified proteins were matched to those in Poaceae like *Oryza sativa, Triticum urartu, Zea mays*, and *Setaria italic*, which are very close to bermudagrass based on gene sequence alignment like dehydration responsive element binding protein (*DREB*) and heat shock protein (*HSP*) (data not shown). The hierarchical cluster (Figure 3D) and venn diagram analyses (Figure 3C) of the significantly changed proteins were performed. The results of hierarchical cluster and venn diagram analysis showed that 33 up-regulated proteins (spots 1, 2, 3, 4, 5, 9, 11, 15, 19, 20, 21, 22, 23, 26, 28, 29, 30, 32, 33, 35, 36, 37, 38, 48, 49, 51, 52, 56, 58, 61, 64, 65, and 72) and 3 down-regulated protein (spots 32, 67, and 71) of 77 identified proteins were simultaneously affected by drought and salt stresses (Figures 3B–D). Among 53 up-regulated proteins, 13 and 7 proteins were specifically induced by drought and salt stresses, respectively. Accordingly, among 36 down-regulated proteins, 27 and 6 proteins were specifically induced by drought and salt stresses, respectively (Figure 3).

**Table 1:** **List of proteins changed under drought (D) and salt (Sa) stress conditions in bermudagrass**.

**Spot**	**Log**_**2**_ **fold changes**		**The**.	**Exp**.	**Score**	**Sequence coverage**	**Homologous protein [species]**	**Class of bin code + subcategory**
**no**.	**D vs. C**	**Sa vs. C**	**Mr(KD)/pI**	**Mr(KD)/pI**		**(%)**		
23	**2.99**	**2.59**	35.2/5.52	77.81/5.38	302	62	ATP synthase beta subunit [*Bouteloua curtipendula*]	[1.1] PS.lightreaction
35	**2.83**	**1.32**	35.3/5.57	13.79/5.9	112	15	Uncharacterized protein LOC100191684 [*Zea mays*]	[1.1] PS.lightreaction
55	**3.05**	**−2.76**	29.4/5.27	47.49/6.06	93	9	ATPase alpha subunit [*Oryza sativa*]	[1.1] PS.lightreaction
17	**2.73**	0.67	25.8/7.63	26.22/5.26	283	15	PsbP family protein [*Oryza sativa*]	[1.1] PS.lightreaction
6	**2.63**	0.10	30.4/5.8	27.07/4.84	149	15	Oxygen evolving protein [*Oedogonium obesum*]	[1.1] PS.lightreaction
77	**−1.01**	**2.62**	20.9/8.20	11.53/6.76	128	29	Cytochrome b6-f complex [*Saccharum hybrid*]	[1.1] PS.lightreaction
8	**−3.29**	**1.10**	27.4/8.85	17.97/5.11	77	9	Oxygen-evolving enhancer protein 2 [*Setaria italica*]	[1.1] PS.lightreaction
71	**−3.21**	**−1.49**	38.8/8.92	34.77/6.89	86	24	Ferredoxin–NADP reductase[*Vitis vinifera*]	[1.1] PS.lightreaction
73	**−3.65**	−0.76	15.2/5.03	17.4/5.59	60	50	ATP synthase [*Eleusine coracana*]	[1.1] PS.lightreaction
74	**−8.13**	0.95	24.2/8.54	42.23/65.54	84	24	Cytochrome b6-f complex iron-sulfur [*Oryza sativa*]	[1.1] PS.lightreaction
34	**−2.08**	**−3.29**	25.9/9.14	12.16/5.85	108	16	Oxygen-evolving enhancer[*Chlamydomonas reinhardtii*]	[1.1] PS.lightreaction
50	**−1.12**	0.91	20.7/6.41	9.34/6.27	80	19	Cytochrome b6-f complex [*Saccharum hybrid*]	[1.1] PS.lightreaction
11	**4.68**	**3.87**	61.2/5.06	66.99/4.98	158	10	RuBisCO binding protein[*Brachypodium distachyon*]	[1.3] PS.calvin cycle
19	**4.08**	**1.10**	53.7/4.88	71.0/5.28	138	15	RuBisCO binding protein[*Secale cereal*]	[1.3] PS.calvin cycle
30	**1.91**	**2.86**	74.0/5.44	72.55/5.51	80	13	Transferase [*Oryza sativa*]	[1.3] PS.calvin cycle
33	**3.01**	**2.88**	11.7/5.41	11.93/5.78	118	26	Chloroplast RuBisCO[*Flaveria trinervia*]	[1.3] PS.calvin cycle
32	**1.20**	**1.09**	15.6/8.24	11.45/5.67	133	16	RuBisCO [*Flaveria palmeri*]	[1.3] PS.calvin cycle
58	**1.28**	**1.26**	51.7/6.14	63.57/6.16	75	24	RuBisCO large subuni [*Bromus inermis*]	[1.3] PS.calvin cycle
5	**4.24**	**2.76**	28.8/5.53	27.78/4.76	114	8	Ribose-5-phosphate isomerase [*Zea mays*]	[1.3] PS.calvin cycle
65	**2.42**	**1.09**	42.9/6.60	34.43/6.84	360	13	Dehydrogenase A[*Setaria italica*]	[1.3] PS.calvin cycle
38	**3.06**	**1.45**	50.9/8.61	45.9/5.82	82	20	RuBisCO activase[*Solanum pennellii*]	[1.3] PS.calvin cycle
16	**2.65**	0.17	32.7/6.96	25.11/5.27	130	11	Triosephosphate isomerase [*Oryza sativa*]	[1.3] PS.calvin cycle
18	**2.82**	0.13	21.7/4.78	42.33/5.36	138	25	RuBisCO activase [*Oryza sativa*]	[1.3] PS.calvin cycle
12	**−2.07**	**1.68**	57.7/4.83	68.26/5.16	135	9	RuBisCO binding protein [*Triticum aestivum*]	[1.3] PS.calvin cycle
13	**−3.11**	**2.29**	52.5/6.14	64.01/5.15	236	26	RuBisCO [*Neyraudia reynaudiana*]	[1.3] PS.calvin cycle
42	**−6.39**	**1.34**	47.5/6.22	44.85/5.85	188	12	glyceraldehyde-3-phosphate dehydrogenase [*Oryza sativa*]	[1.3] PS.calvin cycle
62	**−5.08**	**1.12**	49.3/6.34	28.65/6.62	186	26	RuBisCO large subunit[*Eleusine indica*]	[1.3] PS.calvin cycle
10	**−9.12**	0.30	21.7/4.78	47.95/5.17	213	22	RuBisCO activase [*Oryza sativa*]	[1.3] PS.calvin cycle
41	**−5.06**	0.97	40.5/9.17	48.9/5.68	78	18	Dehydrogenase A[*Chlamydomonas reinhardtii*]	[1.3] PS.calvin cycle
45	**−8.87**	0.42	48.2/6.33	58.6/5.63	176	27	RuBisCO large subunit[*Digitaria radicosa*]	[1.3] PS.calvin cycle
59	**−10.31**	0.21	5.2/5.06	7.77/6.73	107	72	RuBisCO small subunit protein [Eleusine coracana]	[1.3] PS.calvin cycle
75	**−6.16**	−0.10	26.4/7.78	15.13/5.81	170	32	RuBisCO large subunit [*Acorus calamus*]	[1.3] PS.calvin cycle
40	**−1.54**	−0.95	45.2/5.68	72.75/5.67	132	20	Phosphoribulokinase (PRK) [*Oryza sativa*]	[1.3] PS.calvin cycle
43	**−1.02**	0.69	48.0/7.57	42.7/5.80	134	11	RuBisCO activase[*Phaseolus aureus*]	[1.3] PS.calvin cycle
28	**1.21**	**1.02**	48.1/5.17	64.75/5.55	62	15	Enolase 1-like isoform X4 [*Setaria italica*]	[4.1] glycolysis.cytosolic branch
39	**−15.78**	0.99	20.4/8.89	49.39/5.62	349	24	Hypothetical protein ZEAMMB73_319281 [*Zea mays*]	[4.1] glycolysis.cytosolic branch
46	**−11.19**	0.81	60.6/5.49	73.77/5.59	206	15	Phosphoglycerate mutase-like [*Setaria italica*]	[4.1] glycolysis.cytosolic branch
48	**3.89**	**1.97**	48.2/5.59	70.07/5.93	133	18	Enolase 2-like isoform X2 [*Setaria italica*]	[4.1] glycolysis.cytosolic branch
68	**4.08**	**−4.89**	39.0/7.52	41.93/6.83	120	28	Fructose-bisphosphate aldolase [*Zea mays*]	[4.1] glycolysis.cytosolic branch
53	**1.02**	0.78	40.9/7.14	23.44/6.44	92	23	Malate dehydrogenase [*Setaria italica*]	[8.2] TCA / org transformation
66	**1.69**	**−6.33**	36.4/8.88	34.43/6.79	58	19	Malate dehydrogenase[*Citrullus lanatus*]	[8.1] TCA / org transformation.
70	**−2.90**	0.32	46.4/6.34	46.45/6.92	140	30	NADP-isocitrate dehydrogenase [*Oryza sativa*]	[8.1] TCA / org transformation.
25	**−4.02**	0.44	59.5/5.95	15.93/5.5	142	52	ATP synthase subunit beta family protein [*Zea mays*]	[9.9] electron transport / ATP synthesis
31	**2.18**	**−2.24**	55.6/5.70	70.66/5.54	117	17	ATP synthase subunit alpha[*Setaria italica*]	[9.9] electron transport / ATP synthesis
49	**2.67**	**1.28**	17.1/9.97	10/6.37	106	10	Ferredoxin-thioredoxin reductase[*Zea mays*]	[21.1] redox.thioredoxin
36	**2.62**	**1.44**	27.2/5.18	25.26/5.6	77	16	L-ascorbate peroxidase 2 [*Setaria italica*]	[21.2] redox.ascorbate and glutathione
64	**3.40**	**5.64**	17.4/5.56	27.21/6.59	98	22	Hypothetical protein OsI_18213 [*Oryza sativa*]	[21.2] redox.ascorbate and glutathione
1	**2.10**	**1.07**	26.3/6.32	11.65/4.69	81	11	Peroxiredoxin-2E-1 [*Triticum urartu*]	[21.5] redox.peroxiredoxin
63	−0.63	**−2.06**	28.2/5.97	27.72/6.67	89	21	2-Cys peroxiredoxin BAS1 [*Setaria italica*]	[21.5] redox.peroxiredoxin
51	**1.97**	**2.25**	15.3/5.65	11.81/6.32	96	20	Cu/Zn superoxide dismutase [*Aeluropus lagopoides*]	[21.6] redox.dismutases and catalases
69	**−2.77**	−0.39	33.9/9.22	46.68/6.75	69	15	Peroxidase 12 [*Aegilops tauschii*]	[26.12] misc.peroxidases
76	**−6.03**	−0.96	31.2/8.84	28.23/5.92	209	15	Peroxidase 70 [*Aegilops tauschii*]	[26.12] misc.peroxidases
60	**−9.39**	**2.24**	27.8/8.24	13.23/6.65	200	12	50S ribosomal protein L10 [*Aegilops tauschii*]	[29.2] protein.synthesis
26	**2.31**	**1.98**	23.4/6.21	20.8/5.51	123	30	50S ribosomal protein L21 [*Oryza sativa*]	[29.2] protein.synthesis
29	**2.40**	**1.27**	102.1/6.23	81.76/5.52	374	29	Hypothetical protein ZEAMMB73_120778 [*Zea mays*]	[29.5] protein.degradation
7	**2.04**	0.57	46.8/4.82	45.05/4.71	166	14	Peptidyl-prolyl cis-trans isomerase [*Oryza sativa*]	[29.6] protein.folding
44	**−3.19**	−0.03	101.8/6.14	90.3/5,81	278	30	Chaperone protein [*Oryza sativa*]	[29.5] protein.degradation
20	**3.39**	**3.75**	62.1/5.43	60.95/5.37	187	13	Chaperonin 60 subunit beta 2 [*Setaria italica*]	[29.6] protein.folding
4	**2.92**	**3.14**	27.1/6.71	17.43/4.88	167	15	Chitinase *[Leymus chinensis*]	[20.1] stress.biotic
22	**3.56**	**1.55**	71.6/5.11	75.22/5.29	156	29	Heat shock cognate 70[*Petunia hybrid*]	[20.2] stress.abiotic
52	**3.25**	**2.32**	72.4/5.81	17.06/6.17	69	18	Heat shock 70 kDa protein[*Pisum sativum*]	[20.2] stress.abiotic
56	**3.03**	**1.29**	43.2/5.61	47.49/6.19	254	17	S-adenosylmethionine synthase 1 [*Triticum urartu*]	[13.1] amino acid metabolism.synthesis
47	**−6.54**	0.75	22.2/6.74	71.42/5.79	208	36	Alanine aminotransferase [*Zea mays*]	[13.1] amino acid metabolism.synthesis
57	**−8.91**	−0.33	84.7/5.74	82.56/6.08	137	15	Homocysteine methyltransferase [*Setaria italica*]	[13.1] amino acid metabolism.synthesis
67	**−3.48**	**−7.68**	40.8/8.93	33.13/6.90	351	20	Cysteine synthase [*Zea mays*]	[13.1] amino acid metabolism.synthesis
9	**4.36**	**3.23**	45.1/5.51	44.39/4.88	237	28	Unnamed protein product [*Oryza sativa*]	[19.10] tetrapyrrole syn.mg chelatase
15	**2.87**	**2.16**	29.7/6.46	22.68/5.28	102	6	Adenylate kinase, putative [*Ricinus communis*]	[23.4] nucleotide metabolism.kinase
27	**2.72**	0.74	32.8/5.43	28.94/5.5	77	6	Lactoylglutathione lyase [*Aegilops tauschii*]	[24.2] Xenobiotics.lactoylglutathione lyase
37	**2.94**	**1.48**	32.8/5.57	29.03/5.59	133	18	Lactoylglutathione lyase [*Triticum urartu*]	[24.2]Xenobiotics.lactoylglutathione lyase
61	**1.76**	**2.59**	34.7/5.92	29.73/6.88	207	7	Beta-glucanase precursor [*Oryza sativa*]	[26.4] misc.beta 1,3 glucan hydrolases
14	**−3.63**	0.26	26.4/8.67	18.1/5.25	205	47	30S ribosomal protein 2 [*Setaria italica*]	[27.3] RNA.regulation of transcription
3	**1.86**	**2.16**	23.7/4.62	21.41/4.28	162	16	31 kDa ribonucleoprotein [*Triticum urartu*]	[27.4] RNA.RNA binding
24	**3.11**	0.01	15.9/5.52	10.96/5.43	97	30	Glycine-rich protein 1 [*Oryza sativa*]	[27.4] RNA.RNA binding
72	**2.66**	**2.05**	26.6/9.40	25.00/7.00	213	22	Peptidyl-prolyl cis-trans isomerase[*Zea mays*]	[31.3] cell.cycle
21	**3.72**	**1.88**	54.1/5.07	59.73/5.26	311	36	ATPase [Oryza sativa]	[34.1] transport.p- and v-ATPases
2	**4.41**	**1.86**	12.6/8.54	24.23/4.42	68	39	Protein yippee-like protein [*Zea mays*]	[35.2] not assigned.unknown
54	**2.44**	**−8.08**	31.4/9.13	26.24/6.15	127	5	Dehydratase family protein [*Oryza sativa*]	[35.2] not assigned.unknown

*The fold change scales were as following:* 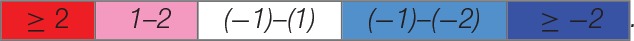

*Legend: Spot no., protein spot number; The., theoretical value; Exp., experimental value; C, control; D, drought; Sa, salt. Different pathway subcategories were highlighted with colors*.

### Pathway enrichment analysis of proteins induced by drought and salt stresses

**Figure 4:**
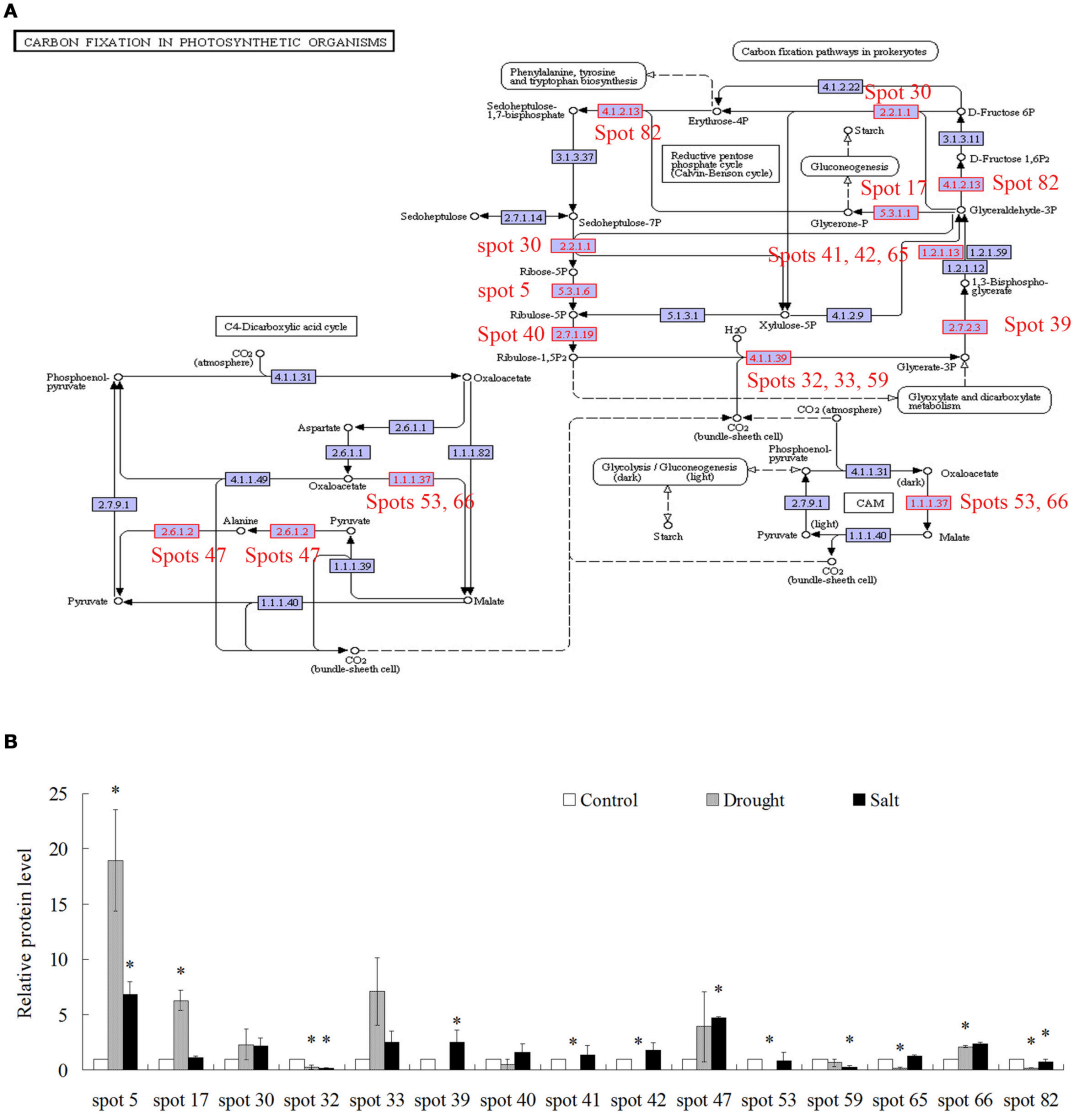
**Proteins whose abundance was changed by treatments of drought and salt were involved in carbon fixation in photosynthetic organisms. (A)** Proteins changed by drought and salt treatments were involved in carbon fixation in photosynthetic organisms. **(B)** The relative protein levels of 15 proteins changed by drought and salt treatments. The results shown are the means ± SE, and the means are the average of three gels from three independent experiments. Asterisk symbols (^*^) indicate *P* < 0.05 (*t*-test).

Because of limited reference genome information for bermudagrass, the homologous proteins were blasted against sequenced plant species and functional categories were also assigned using MapMan. The information of homologous protein and functional category of each protein was shown in Table 1 and Table S1. The MapMan pathway enrichment analysis revealed that functions of commonly regulated proteins by drought and salt stresses were involved in metabolism pathways including photosynthesis (PS), biodegradation of xenobiotics, oxidative pentose phosphate pathway (OPP), glycolysis and redox (Table 2, Group I). Other pathways including N-metabolism, TCA/org transformation, amino acid metabolism, and mitochondrial electron transport/ATP synthesis were specifically affected by drought or salt stress (Table 2, Group II). Additionally, 15 proteins were involved in carbon metabolic pathway (Figure 4A). Abundances of these proteins were increased or decreased in response to drought and salt stresses. For example, the abundance of protein spot 5 was significantly induced by drought and salt stresses, while that of protein spot 57 was significantly decreased after drought treatment (Figure 4B).

**Table 2:** **Pathway enrichment analysis of proteins changed by drought and salt treatments in bermudagrass**.

**Group**	**MapMAN pathways**	**Drought**		**Salt**	
		**NF**	***P*-value**	**NF**	***P*-value**
I	PS	**62.24**	0.0000	**54.37**	0.0000
	Biodegradation of Xenobiotics	**31.58**	0.0018	**44.44**	0.0009
	OPP	**28.52**	0.0022	**40.14**	0.0011
	Glycolysis	**27.98**	0.0000	**23.63**	0.0003
	Redox	**14.73**	0.0000	**17.77**	0.0000
II	N-metabolism	**17.00**	0.0560	**23.93**	0.0400
	TCA / org transformation	**16.78**	0.0007	**7.87**	0.1120
	Amino acid metabolism	**6.80**	0.0026	**9.57**	0.0008
	Mitochondrial electron transport/ATP synthesis	**5.85**	0.0410	**4.12**	0.1910
III	Nucleotide metabolism	**2.45**	0.2730	**3.45**	0.2180
	Tetrapyrrole synthesis	**9.21**	0.0980	**12.96**	0.0720
	Stress	1.08	0.2270	1.52	0.1810
	Misc	0.83	0.2170	0.78	0.2580
	Protein	0.73	0.0890	0.77	0.1320
	RNA	0.58	0.0920	0.40	0.0840
	Cell	0.53	0.2880	0.74	0.3550
	Transport	0.43	0.2280	0.60	0.3200
	Not assigned	0.07	0.0000	0.10	0.0000

*NF, normalized frequency of each functional category in genome; PS, photosynthesis; OPP, oxidative pentose phosphate pathway. Black background means NF > 2 and P < 0.05 and gray background means NF > 2 while P > 0.05*.

### Modulation of ROS metabolism in bermudagrass after drought and salt treatments

**Figure 5:**
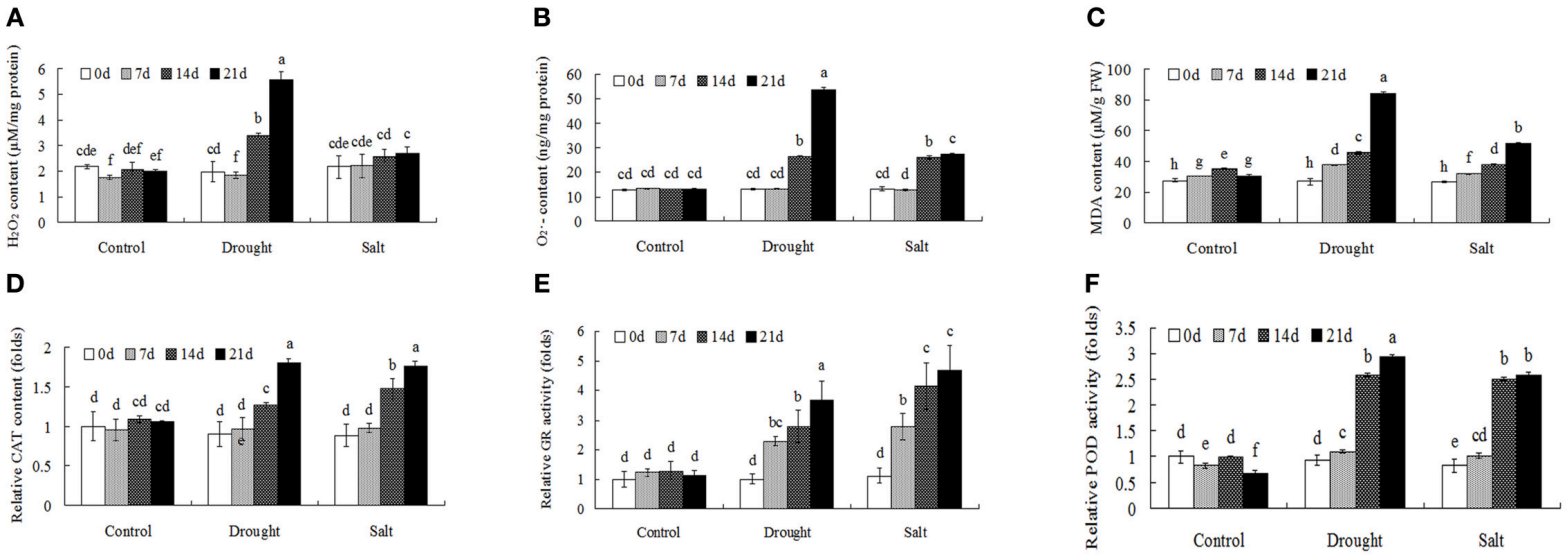
**ROS level and antioxidant enzyme activities of bermudagrass under control and stress conditions at designated time intervals**. Changes of H_2_O_2_ content **(A)**, ${\text{O}}_{2}^{•-}$ content **(B)**, and MDA level **(C)** of bermudagrass under control and stress conditions at indicated days. **(D–F)** Comparisons of CAT **(D)**, GR **(E)**, and POD **(F)** activities of bermudagrass under control and stress conditions at designated time intervals. The relative activities were quantified as fold change in comparison with bermudagrass under unstress condition. The data represent the means of three independent experiment ± SE, and data followed by different letters are significantly different from each other at *P* < 0.05 according to Duncan's method.

Through proteomics approach we identified several ROS metabolism related proteins in bermudagrass after drought and salt treatments, including POD (protein spot 36, 69, and 76), SOD (protein spot 51) and peroxiredoxin (protein spot 1 and 63). To further investigate ROS homeostasis caused by drought and salt stresses, H_2_O_2_, ${\text{O}}_{2}^{•-}$ and MDA contents were analyzed (Figures 5A–C). In terms of H_2_O_2_, ${\text{O}}_{2}^{•-}$, and MDA, drought stress caused significantly higher levels at the end of experiment in comparison to salt treatment (Figures 5A–C). In addition, the levels of H_2_O_2_, ${\text{O}}_{2}^{•-}$, and MDA were progressively induced by salt stress, especially at 21 days after treatment (Figures 5A–C).

Antioxidant enzymes activities, including CAT, GR, and POD, were then analyzed to reveal changes of enzymatic defense systems (Figures 5D–F). When exposed to drought and salt conditions, the activities of CAT, GR, and POD had similar changes, which were largely induced with the duration of the treatments. (Figures 5D–F).

### Modulation of metabolic homeostasis in response to drought and salt stresses

**Figure 6:**
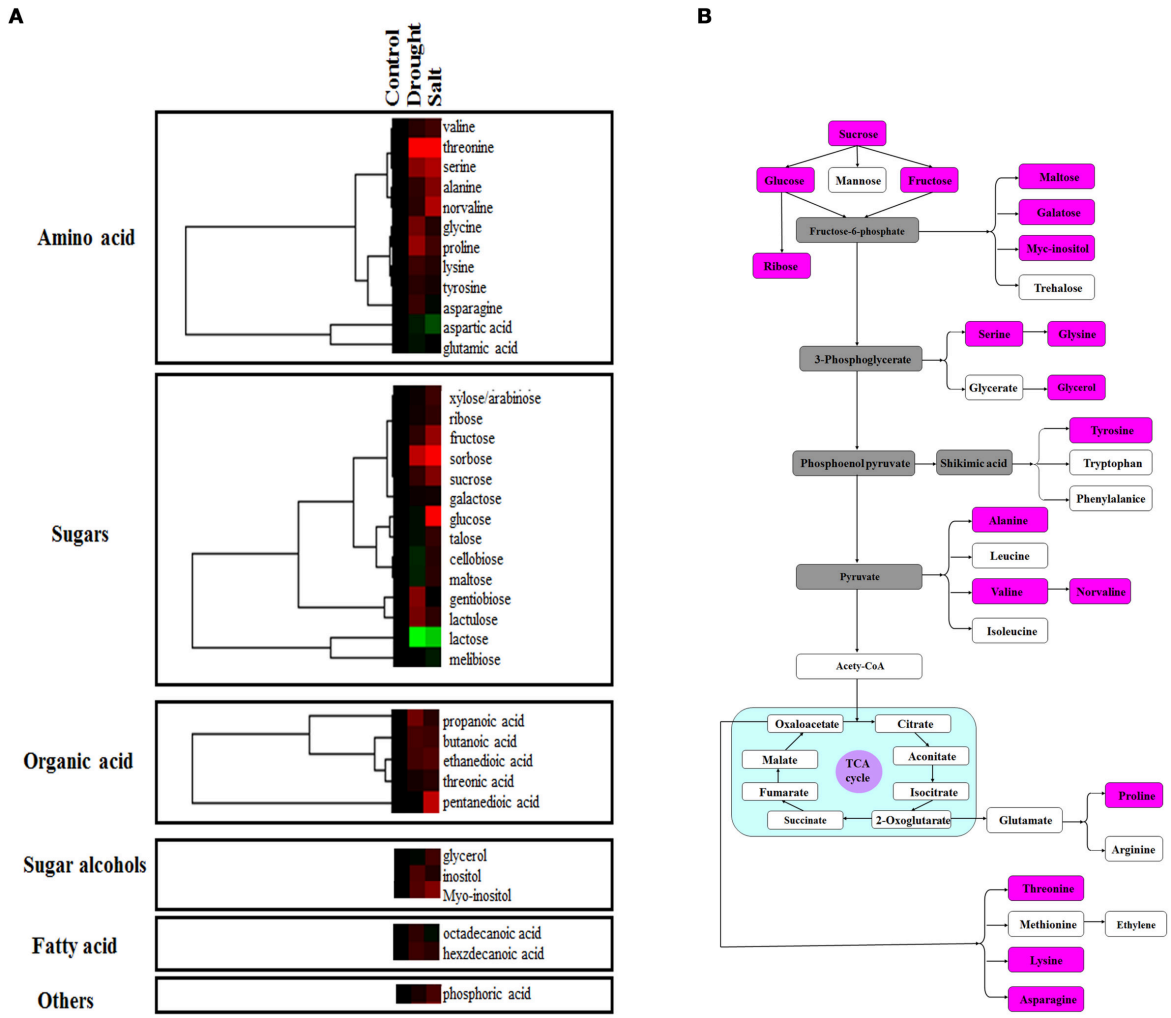
**Effects of drought and salt stresses on metabolites in bermudagrass. (A)** Hierarchical cluster analysis of 37 compounds affected by drought and salt stress conditions in bermudagrass. The resulting tree figure was obtaining using the Java Treeview and the CLUSTER software package. The metabolites that were shown in panel **(B)** were indicated with rose red colors. **(B)** Assignment of the 18 metabolites studied to pathways. A total of 18 metabolites were indicated in boxes with rose red colors, and the concentrations of these metabolites were shown in Table S2.

Proteomic analysis indicated that functions of several proteins were involved in carbon fixation pathway (Figure 4A). To further reveal the modulation of metabolic homeostasis in bermudagrass regulated by drought and salt stresses, metabolomic analyses based on chromatography time-of-flight mass spectrometry (GC-TOF-MS) were performed using 14 days stressed samples. The results showed that totally 37 metabolites, including 12 amino acids, 14 sugars, 5 organic acids, 2 sugar alcohols, and 2 fatty acid were successfully detected (Figure 6A, Table S2). Generally, 30 of 37 metabolites were reproductively detected after drought and salt treatments (Figure 6A and Table S2). Interestingly, contents of most metabolites increased after drought and salt stress treatments. Several metabolites including threonine, serine, propanoic acid, and ethanedioic acid simultaneously showed higher level by drought and salt, whereas lactose concentration simultaneously decreased by two kinds of stress (Figure 6A). Among 37 metabolites, 18 metabolites involved in carbon and amino acid metabolic pathways (Figure 6B) were commonly modulated by drought and salt stresses, further confirming the carbon and amino acid metabolisms were extensively changed in response to abiotic stresses.

## Discussion

Drought and salt are common stress conditions that severely affect plant growth and crop production. In this study, comparative analyses of bermudagrass in response to drought and salt stresses at physiological, proteomic, and metabolomic levels were performed. The results provided evidences that bermudagrass showed both common and contrasting responses to drought and salt treatments.

### Cell membrane damages caused by drought and salt

In plant cells, osmotic stress caused by drought and salt conditions leads to cell membrane damages. EL is a key parameter to assess cell membrane stability in response to water stress. Although drought and salt treatments caused increased EL and membrane damage (Figure 1E), salt stress showed less effect on EL and plant survival rate (Figures 1E,F) indicating that bermudagrass was highly tolerant to 400 mM NaCl within 21 days treatment which was consistent with previous studies (Shi et al., 2013a,b; Lu et al., 2007). In bermudagrass variety “WBD128” collected in China, 200 mM NaCl treatment for 5 days resulted in a slight increase of EL when compared to the control (Liu et al., 2016). These results indicated that several bermuagrass varieties are salt tolerant and salt stress caused slight cell membrane damages in these bermudagrass varieties.

### Modulation of photosynthesis and carbon fixation by drought and salt

Comparative proteomic analyses suggested that abundance of 33 proteins commonly increased and abundance of 3 proteins commonly decreased by both drought and salt (Figures 3, 4). Among them, the majority of proteins involved in PS and carbon fixation pathways were commonly regulated by drought and salt stresses. Ribulose-1, 5-bisphosphate carboxylase/oxygenase (Rubisco) is a predominant protein in photosynthesizing parts of plant and considered as the most important protein in PS. As reported, Rubisco efficiency is closely involved in plants abiotic stress responses (Feller et al., 2008). In this study, 7 RuBisCO-related proteins (spots 11, 19, 32, 33, 38, 58, 75), 3 other proteins involved in calvin cycle (spots 5, 30, 650), and 2 proteins involved in light reaction (spots 23, 35) were commonly activated by both drought and salt stresses (Table 1). However, 7 other RuBisCO-related proteins (spots 10, 12, 13, 43, 45, 59, 62) were mainly inhibited by drought, but activated or showed no changes by salt (Table 1). After water deficit, the quantity of RuBP and photorespiration in the leaves declined in three different C4 grasses, including bermudagrass (Carmo-Silva et al., 2008, 2010). All these data showed that proteins involved in light reaction and carbon fixation were partially impressed by drought, but mainly activated by salt. These results were in agreement with changes of metabolites content, which showed that sugars and sugar alcohols increased significantly by salt, but most of them slightly by drought (Figure 6). Therefore, both proteomic and metabolomic approaches indicated that drought stress caused complex changes on carbon fixation, while salt stress increased carbonhydrate biosynthesis. Soluble sugars and sugar alcohols function as compatible solutes which are responsible for osmotic balance and protect protein and other macromolecules from stress caused damages (Zhu, 2002). Accumulation of compatible solutes under salt condition enhanced bermudagrass tolerance to salt stress, while under drought condition, relative lower compatible solutes content resulted in more severe cell membrane damages as evidenced by increased EL and decreased survival rate in bermudagrass (Figures 1E–F).

### Modulation of energy supply by drought and salt

Proteomic analysis showed that several proteins (spots 25, 31) involved in photosynthesis electron transport were inhibited by drought and salt stresses in bermudagrass, indicating that the modulation of these proteins might be directly relative with drought and salt tolerance signaling pathway in bermudagrass (Yang et al., 2003). Ferredoxin-thioredoxin reductase (spot 49) and ferredoxin-NADP reductase (spot 71) were differentially induced by drought and salt treatments. Chloroplast ferredoxin acted as a redox protein in photosynthesis participates in both cyclic and non-cyclic photophosphorylation reactions (Yang et al., 2003). Ferredoxin is the last electron acceptor and reduces the enzyme NADP^+^ reductase in non-cyclic photophosphorylation (Fukuyama, 2004). These results showed the energy supply adjustments in bermudagrass under abiotic stress conditions.

Moreover, glycolysis provides energy such as ATP and NADH and generates precursors for anabolism such as fatty acids and amino acids (Plaxton, 1996; Shi et al., 2014c). In present study, five proteins (spots 28, 39, 46, 48, 68) involved in glycolysis metabolic pathway were differentially affected when exposed to drought and salt stresses. For example, the abundance of glyceraldehyde 3-phosphate dehydrogenase (GAPDH, spots 41, 42) increased by salt stress, but decreased by drought. Cytosolic GAPDH was induced by abiotic stresses and overexpression of *GAPDH* increased plant abiotic stress tolerances (Zhang et al., 2011; Guo et al., 2012). In addition, the abundance of fructose-bisphosphate aldolase (spot 68) and malate dehydrogenase (spots 53, 66) was significantly increased by drought, but inhibited by salt stress. Moreover, one ATP synthase subunit alpha (spot 31) was enhanced by drought but inhibited by salt, while an ATPase (spot 21) was activated by both drought and salt (Table 1). Based on RNA seq data, Hu et al. (2015) identified 536 and 848 differentially expressed genes in two bermudagrass varieties after salt treatment. Genes involved in ATP catabolic process pathway were downregulated by salt. These data indicated that metabolisms for energy supply were more extensively affected by drought than by salt in bermudagrass, resulting in faster energy production and more quick carbohydrate degradation. This hypothesis was consistent with content changes of sugars and sugar alcohols as detected by GC-TOF-MS (Figure 6).

### Redox and stress related proteins modulated by drought and salt

When exposed to stress conditions, plants often suffer oxidative damages due to ROS accumulation (Morita et al., 2011). Protection against oxidative damages is a key component of stress tolerance in plants (Alscher et al., 2002; Atamna and Boyle, 2006). In this study, several antioxidant enzymes including POD (spots 36, 69, 76) and superoxide dismutase (SOD) (spot 51) were regulated by drought and salt stresses. Physiological analysis also showed that both drought treatment resulted in accumulation of H_2_O_2_ and ${\text{O}}_{2}^{•-}$ contents, while 400 mM salt treatment only increased ${\text{O}}_{2}^{•-}$ contents. Both drought and salt increased activities of CAT, POD, and GR (Figure 5). These results were in line with protein abundance changes through proteomics approach (Table 1). ROS plays key roles during plant abiotic stress response. In response to abiotic stress condition, Shi et al. (2012) and Liu et al. (2016) also found that drought or salt stress induced ROS production and increased activities of antioxidant enzymes, including CAT, SOD, and POD. Accumulation of ROS might act as stress signaling in bermudagrass which in turn activated downstream stress responsive genes.

Several other proteins (spots 7, 20, 26, 29, 44, and 60) related to the protein folding, protein synthesis and protein degradation were regulated by drought and salt stresses. For example, the ribosomal protein (spots 26, 60) involved in the cellular process of translation (Balmer et al., 2003) was commonly regulated by drought and salt. Moreover, one chitinase (spot 4), and two heat shock protein 70 (spots 22 and 55) were commonly induced by drought and salt, which play pivotal roles in plant stress responses (Wang et al., 2004; Dana et al., 2006), indicating that bermudagrass sensed environmental stress signaling and responded to stress conditions through modulation of stress responsive genes. Most recently, overexpression of bermudagrass genes *CtHsfA2b* and *NF-YC* improved abiotic stress tolerance in Arabidopsis and rice, respectively (Chen et al., 2015; Wang et al., 2016). These results indicated that candidate genes from bermudagrass might be effective to improve stress tolerance in model and crop plants. Since several bermudagrass varieties showed high tolerance to drought and salt, identification and functional characterization of stress responsive genes would provide new gene resources for genetic modification of bermudagrass with increased stress tolerance.

**Figure 7:**
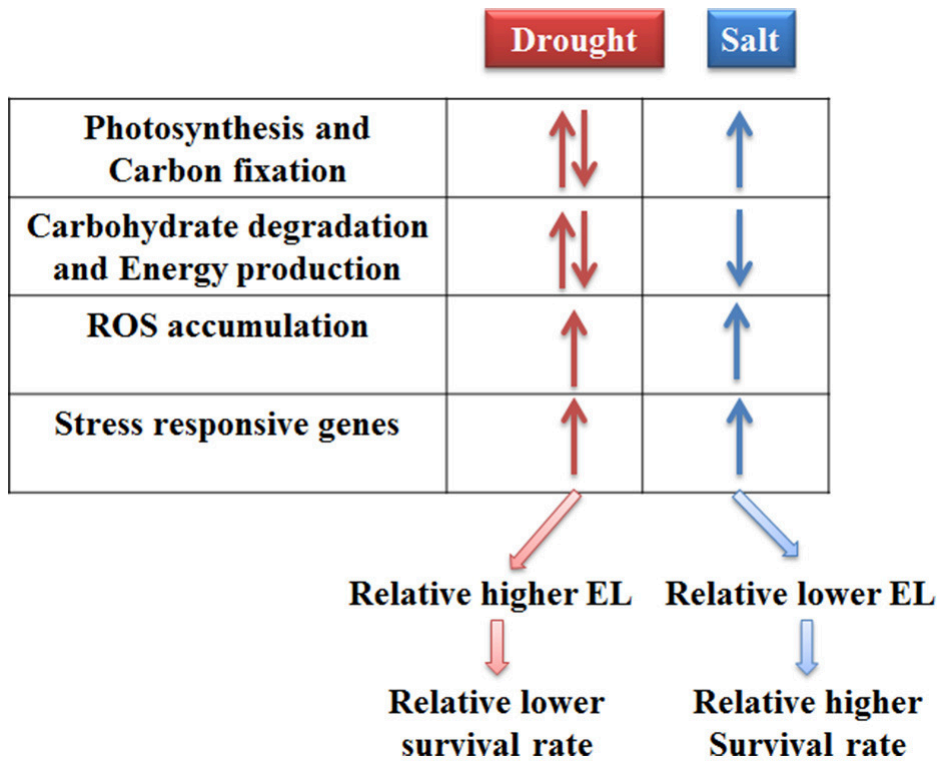
**A proposed model for drought and salt stress responses in bermudagrass**.

Taken together, the responses of bermudagrass to drought and salt stresses were compared at proteomics and metabolomics levels. The results indicated that photosynthesis and carbon fixation pathways were extensively affected under both drought and salt conditions. Drought stress caused relatively less carbohydrate accumulation and more energy production which might result in carbon starvation. Conversely, salt stress induced sugars and sugar alcohols accumulation which functioned as compatible solutes. Moreover, drought and salt stresses increased ROS levels and modulated proteins involving in stress tolerance in bermudagrass. Therefore, 21 days drought treatment finally led to relatively more severe cell membrane damage and lower survival rate in relative to 400 mM NaCl treatment (Figure 7).

## Author contributions

TY designed and performed the experiments, analyzed the data, and wrote the manuscript; HS performed the experiments, analyzed the data, and revised the manuscript; YW analyzed the data; FY revised the manuscript; ZC designed the experiments, and revised the manuscript; and all authors approved the manuscript.

### Conflict of interest statement

The authors declare that the research was conducted in the absence of any commercial or financial relationships that could be construed as a potential conflict of interest.

## Abbreviations

APX: ascorbate peroxidase
CAT: catalase
2-DE: two-dimensional electrophoresis
EL: electrolyte leakage
FBAs: Fructose-1,6-bisphosphate aldolases
GAPDH: gyceraldehyde 3-phosphate dehydrogenase
GR: glutathione reductase
IEF: isoelectric focusing
IPG: immobilized protein gradient
RWC: relative leaf water content
MDA: malondialdehyde
NF: normalized frequency
OPP: oxidative pentose phosphate pathway
OEE2: oxygen-evolving enhancer protein 2
POD: peroxidase
PRK: phosphoribulokinase
PS: photosynthesis
ROS: reactive oxygen species
Rubisco: ribulose-1,5-bisphosphate carboxylase/oxygenase
RuBP: ribulose-1,5-bisphosphate
SOD: superoxide dismutase
TCA: tricarboxylicacid
WAK1: wall-associated receptor kinase.
