# Supplementary figures and images for: Contrasting Proteomic and Metabolomic Responses of Bermudagrass to Drought and Salt Stresses
Source: Front Plant Sci. 2016 Nov 11;7:1694. doi: 10.3389/fpls.2016.01694 (PMC5105639; doi:10.3389/fpls.2016.01694)

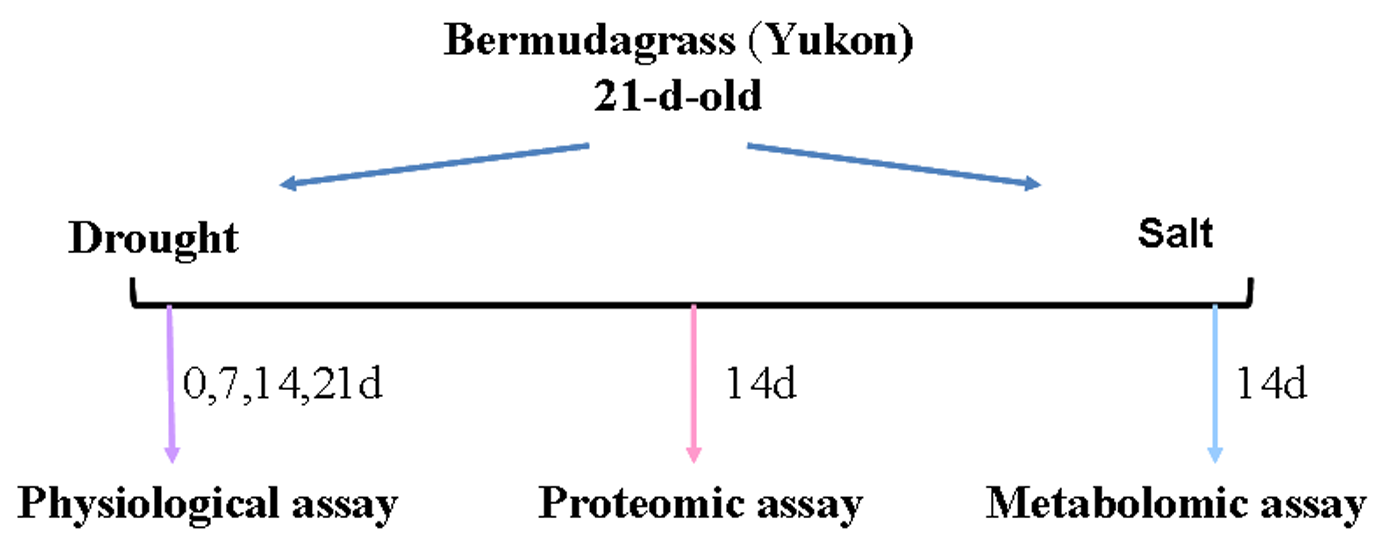

Supplement: Supplementary file 1 [file Image1.TIF]
